# Characterization of the complete mitochondrial genome of the *Libelloides sibiricus* (Neuroptera, Ascalaphidae)

**DOI:** 10.1080/23802359.2024.2339486

**Published:** 2024-04-12

**Authors:** Qingbin Zhan, Yunpeng Gai, Yang Zhao

**Affiliations:** a Department of Criminal Science and Technology, Nanjing Police University, Nanjing, Jiangsu, China; b Key Laboratory of State Forestry and Grassland Administration on Wildlife Evidence Technology, Nanjing, Jiangsu, China; c School of Grassland Science, Beijing Forestry University, Nanjing, Jiangsu China; d Research Institute of Pomology, Nanjing Institute of Agricultural Sciences in Jiangsu Hilly Area, Nanjing, Jiangsu, China

**Keywords:** Owlfly, phylogenetic analysis, mitochondrial genome

## Abstract

*Libelloides sibiricus* (Eversmann, 1850) is widely distributed in China, Korea and eastern Russia. To date, few studies have been conducted on this species, with the exception of morphological taxonomy studies. In this study, we sequenced the complete mitochondrial genome (mitogenome) of *Libelloides sibiricus*, which is 15,811 bp in length, with an overall A + T content of 74.8%, encoding 2 ribosomal RNA genes, 22 transfer RNA genes, 13 protein-coding genes, and a control region. The gene arrangement and components of *L. sibiricus* are identical to those of most other Neuropteran species. TAA is utilized as the termination codon for most PCGs and TAG for *nd1*, however, *nd6* and *atp6* used the incomplete termination codon TA- and *cox1*, *cox2*, *nd5*, *cytb* had termination codons consisting of only T–. In addition, we selected all known 59 species of Neuroptera from NCBI, and used *Sialis hamata*, *Sialis melania*, *Sialis longidens* and *Sialis jiyuni* (Megaloptera: Sialidae) as the outgroup. Phylogenetic analysis suggested that the mitogenome of *L. sibiricus* was the most closely related to *L. macaronius* and all the owlflies formed the monophyletic group within the superfamily Myrmeleontoidea.

## Introduction

Owlfly, which is the common name for the insect group known as Ascalaphidae, has large adult individuals with long, elongated antennae that are enlarged at the tips. The larvae, on the other hand, are very flat and have branched spines and decorative hairs on their body surface. In 1842, Lefèbvre established the family Ascalaphidae, which includes two subfamilies: Holophthalminae and Ascalaphinae. In recent years, there have been significant changes to the understanding of the phylogenetic status of the family Ascalaphidae (Jones 2019; Machado et al. 2019). It proposes that the family Ascalaphidae may actually be a subfamily within the larger family Myrmeleontidae. These revised taxonomic considerations shed new light on the evolutionary history of Ascalaphidae and provide an opportunity for a comprehensive reevaluation of its classification. Future studies can now focus on exploring the genetic relationships and characteristics of these insects to confirm and refine these taxonomic revisions. However, to date, only five owlflies species mitochondrial genomes have been fully sequenced. There are *Libelloides macaronius* (NC_015609), *Ascaloptynx appendiculatus* (FJ171324), *Ascalohybris subjacens* (KC758703), *Ogcogaster segmentator* (ON243766), *Suhpalacsa longialata* (MH361300). The genus *Libelloides* , a member of the family Ascalaphidae, subfamily Ascalaphinae includes 16 species and subspecies occurring in Palacarctic region (Háva 2000; Háva and Ábrahám 2014, Aspöck et al. 2001). In this study, we aimed to sequence and characterize the entire mitogenome of *Libelloides sibiricus* to provide a complete mitogenome reference that is valuable for determining robust phylogenetic relationships and population genomics in owlflies.

## Materials and methods

**Figure 1.**
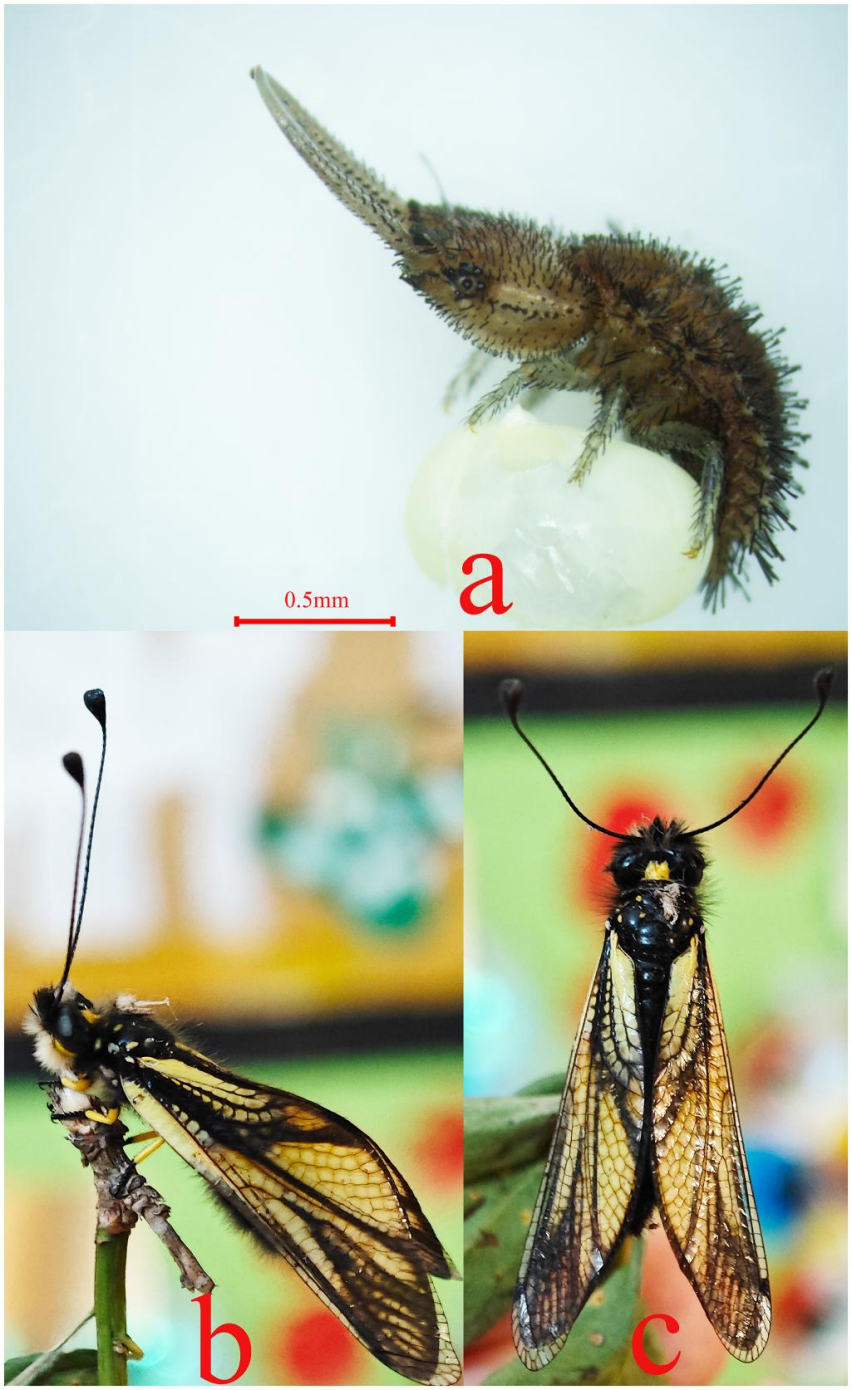
Morphological photograph of *L. sibiricus* (photographed by Yang Zhao). 1a. egg and 1^st^ larve; 1b, c. lateral and dorsal view of adult, respectively.

The material of *Libelloides sibiricus* (Figure 1a,b,c) was collected from Xianghetun, Gaojiadian Town, Xifeng County, Liaoning Province (Geographic location: 42°67'13"N, 124° 45'92" E), China, on 26 May. 2023. The sample was alive during the collection and the specimen was deposited in the Museum of Nanjing Police University under the voucher number NFPC0146 (Qingbin Zhan, zhanqb@nfpc.edu.cn). Total genomic DNA was extracted from thoracic muscle of adult moths using the cetyltrimethylammonium bromide (CTAB) method (Shahjahan et al. 1995). The DNA quality was examined with agarose-gel electrophoresis, and the concentration was measured using Nanodrop. Paired-end libraries with insert sizes of 180 bp were prepared from 1 mg DNA of genomic using Illumina ‘s Genomic DNA Sample Preparation kit (Illumina, San Diego, CA, USA), following the manufacturer’s instructions. Subsequently, the DNA libraries were sequenced using 150 bp paired reads on the Illumina NovaSeq 6000 platform (Illumina, San Diego, CA). Fastp was used to check the quality of data (Chen et al. 2018). A total of 50,504,036 clean reads were generated. Quality control standards are as follows: first, the reads with unrecognized nucleotides >10% were removed. Second, removing reads with >50% bases having Phred quality <5, and the readings aligned with the adapter greater than 10 nt were removed, allowing mismatches ≤ 10%. Finally, the assumed PCR repeats generated by PCR amplification during library construction were removed (the same readings 1 and 2 in the two paired end readings). The mitochondrial genome of *L. sibiricus* was assembled using Novoplasty v2.7 (Nicolas et al. 2016). The MITOS2 web server (Bernt et al. 2013) was utilized to annotate the complete mitogenome of *L. sibiricus*. The tRNA genes were annotated using tRNAscan-SE software (Lowe and Eddy 1997), To ensure accuracy, the annotation errors in the mitochondrial genome were manually corrected using Apollo software[Lewis et al. 2002]. The drawing of mitogenome map was completed by Proksee (Grant et al. 2023).

To investigate the phylogenetic position of *L. sibiricus*, we selected all known 59 species sequences of Neuroptera from NCBI, and used *Sialis hamata*, *Sialis melania*, *Sialis longidens* and *Sialis jiyuni* (Megaloptera: Sialidae) as the outgroup. The protein-coding sequences were aligned using MAFFT version 7.313 (Katoh and Standley 2013). After removing gaps and ambiguous sites using trimAI (Capella-Gutiérrez et al. 2009), we concatenated the sequences using PhyloSuite version 1.2.2 (Zhang et al. 2020). A phylogenetic tree was constructed based on the mitochondrial genome, including 13PCGs and 2 rrna dataset, and the maximum-likelihood (ML) method with the best-fit model estimated using IQ-TREE v1.6.10 (Nguyen et al. 2015). Finally, 1000 bootstrap replicates were used, and the phylogenetic trees were visualized and annotated using the Interactive Tree of Life (ITOL) (https://itol.embl.de/).

## Results

**Figure 2.**
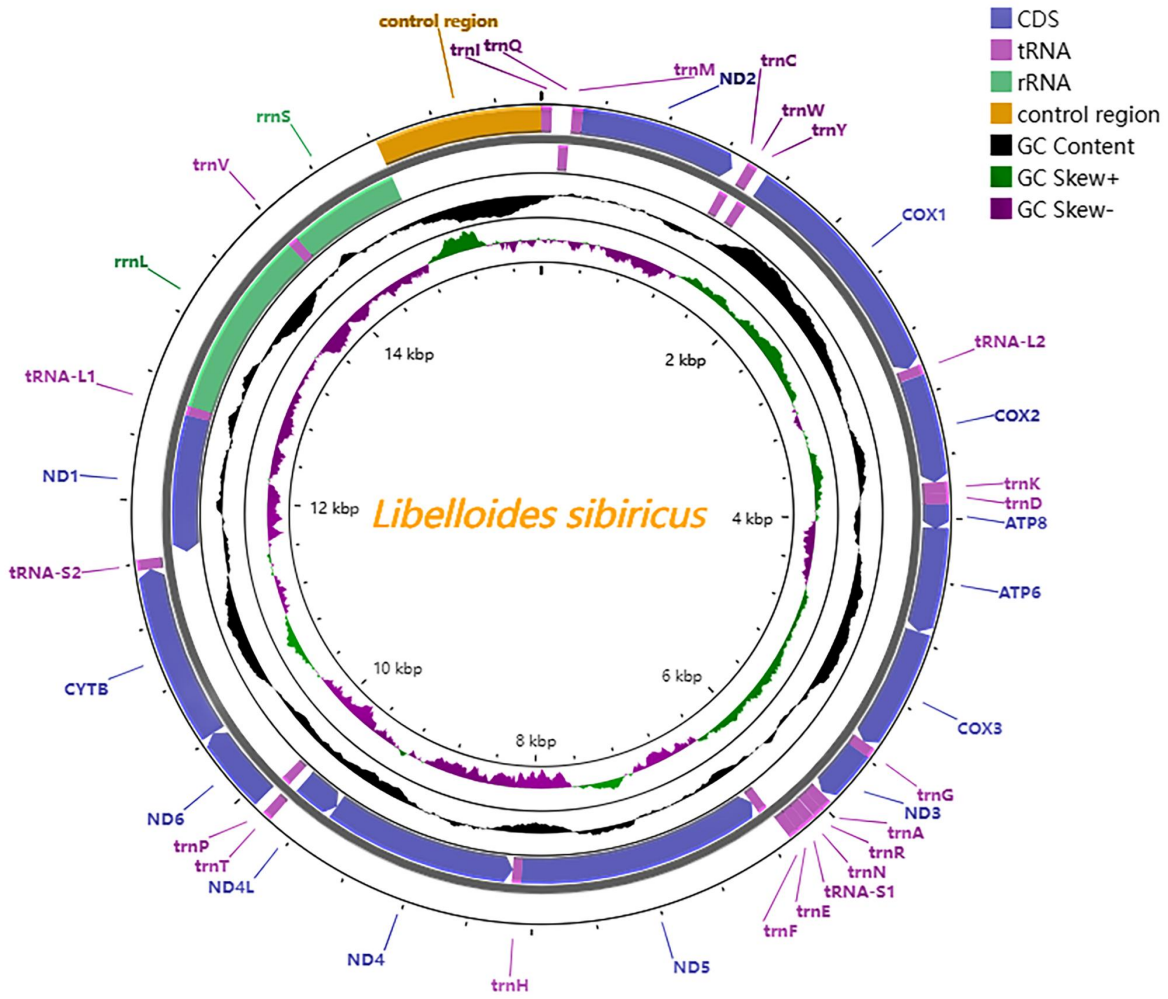
Circular map of the mitochondrial genome of *L. sibiricus.* Genes outside the circle are encoded on the heavy strand and genes inside the circle are encoded on the light stand. The inner black bars indicate the GC content, and the Middle line represents 50%. visualization was performed using proksee (Grant et al. 2023).

The complete circular mitogenome of *L. sibiricus* presented 15881 bp in size (GenBank accession no. OR571471). It contains 37 genes (13 PCGs, 22 tRNA genes, and two rRNA genes) and a control region (Figure 2). The mitogenome sequence has a high AT content of 74.8%. In addition, the majority coding strand (J-strand) contains 23 genes (nine PCGs and 14 tRNA genes) and the minority coding strand (N-strand) contains 14 genes (four PCGs, eight tRNA genes, and two rRNA genes). Analysis of the PCGs showed that the total length of the 13 PCGs was 11,177 bp, of which the *nad5* gene was the longest of 1732 bp and *atp8* was the shortest of 159 bp. All 13 PCGs initiated with the standard start codons ATN, excpt *cox1* with ACG (Table 1). Whereas seven PCGs terminated with complete stop codons (i.e.TAA and TAG), *nd6* and *atp6* use the incomplete termination codon TA- and *cox1*, *cox2*, *nd5*, *cytb* used a single T– residue as the stop codon. The analysis of rRNA genes showed that the length of 16S rRNA was 1315 bp with the AT content of 78.1%, and the length of 12S rRNA was 776 bp with the AT content of 74.7%.

**Table 1.** Mitochondrial genome characteristics of *Libelloides sibiricus.*

Gene	Location	Size (bp)	Strand	Start codon	Stop codon	Intergenic length
*trnI(gau)*	1–66	66	+			
*trnQ(uug)*	117–185	69	–			50
*trnM(cau)*	195–262	68	+			9
*ND2*	263–1276	1014	+	ATC	TAA	0
*trnC(gca)*	1275–1338	64	–			−2
*trnW(uca)*	1346–1413	68	+			7
*trnY(gua)*	1415–1482	68	–			1
*COX1*	1485–3018	1534	+	ACG	T	2
*trnL(uaa)*	3019–3083	65	+			0
*COX2*	3086–3767	682	+	ATG	T	2
*trnK(cuu)*	3768–3838	71	+			0
*trnD(guc)*	3839–3906	68	+			−0
*ATP8*	3907–4065	159	+	ATT	TAA	0
*ATP6*	4059–4735	677	+	ATG	TA	−7
*COX3*	4736–5524	789	+	ATG	TAA	0
*trnG(ucc)*	5524–5588	65	+			−1
*ND3*	5589–5942	354	+	ATA	TAA	0
*trnA(ugc)*	5947–6015	69	+			4
*trnR(ucg)*	6016–6081	66	+			0
*trnN(guu)*	6084–6150	67	+			2
*trnS(gcu)*	6151–6217	67	+			0
*trnE(uuc)*	6220–6284	65	+			2
*trnF(gaa)*	6283–6348	66	–			−2
*ND5*	6349–8080	1732	–	ATT	T	0
*trnH(gug)*	8081–8144	64	–			0
*ND4*	8145–9485	1341	–	ATG	TAA	0
*ND4L*	9486–9773	288	–	ATG	TAA	0
*trnT(ugu)*	9776–9839	64	+			2
*trnP(ugg)*	9840–9905	66	–			0
*ND6*	9907–10424	518	+	ATA	TA	1
*CYTB*	10425–11559	1135	+	ATG	T	0
*trnS(uga)*	11560–11627	68	+			0
*ND1*	11648–12601	954	–	ATA	TAG	20
*trnL(uag)*	12602–12664	63	–			0
*l-rRNA*	12665–13979	1315	–			0
*trnV(uac)*	13980–14050	71	–			0
*s-rRNA*	14051–14826	776	–			0

**Figure 3.**
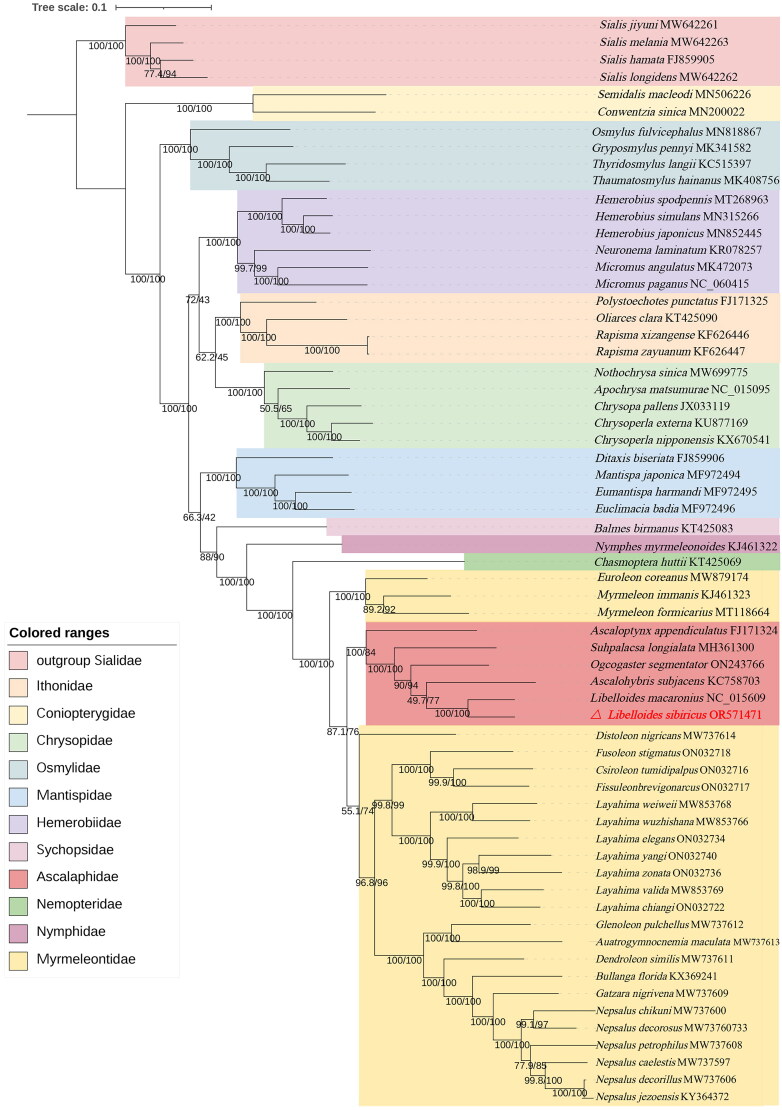
Based on the PCG12 matrix, the phylogenetic tree was reconstructed under the maximum-likelihood (ML) methods, with branch support values denoted SH-Alrt/UFBoot2. The mitochondrial genomes of 59 species of neuroptera and four outgroup species of Sialidae (*S. hamata*, *S. melania*, *S. longidens* and *S. jiyuni*) were selected. Mitochondrial genome sequences are derived from the following records: *Ascalohybris subjacens* KC758703 (Cheng et al. 2014); *Suhpalacsa longialata* MH361300 (Gao et al. 2018); *Ogcogaster segmentator* ON243766 (Wu et al. 2022); *Libelloides macaronius* NC_015609 (Negrisolo et al. 2011); *L. sibiricus* (this study); *Ascaloptynx appendiculatus* FJ171324 (Beckenbach and Stewart 2009); *Myrmeleon formicarius* MT118664 (Wu et al. 2020); *M. immanis* KJ461323 (Yan et al. 2014); *Bullanga Florida* KX369241 (Lan et al. 2016); *Epacanthaclisis banksi* KF701327 (Cheng et al. 2015); *Distoleon nigricans* MW737614 (Zheng et al. 2022a, 2022b); *Austrogymnocnemia maculata* MW737613 (Zheng et al. 2022a, 2022b); *Glenoleon pulchellus* MW737612 (Zheng et al. 2022a, 2022b); *Dendroleon similis* MW737611 (Zheng et al. 2022a, 2022b); *Gatzara nigrivena* MW737609 (Zheng et al. 2022a, 2022b); *Nepsalus petrophilus* MW737608 (Zheng et al. 2022a, 2022b); *N. decorosus* MW737607 (Zheng et al. 2022a, 2022b); *N. decorillus* MW737606 (Zheng et al. 2022a, 2022b); *N. caelestis* MW737597(Zheng et al. 2022a, 2022b); *N. chikuni* MW737600 (Zheng et al. 2022a, 2022b); *Layahima yangi* ON032740 (Zheng et al. 2022b); *L. zonata* ON032736 (Zheng et al. 2022b); *L. chiangi* ON032722 (Zheng et al. 2022b); *L. elegans* ON032734 (Zheng et al. 2022b); *L. valida* MW853769 (Zheng et al. 2022b); *L. weiweii* MW853768 (Zheng et al. 2022b); *L. wuzhishana* MW853766 (Zheng et al. 2022b); *Euroleon coreanus* MW879174 (Guan et al. 2021); *Nepsalus jezoensis* KY364372 (Zhang et al. 2017); *Fusoleon stigmatus* ON032718(Zheng et al. 2023); *Fissuleon brevigonarcus* ON032717 (Zheng et al. 2023); *Csiroleon tumidipalpus* ON032716 (Zheng et al. 2023); *Neuronema laminatum* KR078257 (Zhao et al. 2015); *Hemerobius japonicus* MN852445 (Zhao et al. 2020a); *H. simulans* MN315266; *H. spodipennis* MT268963 (Zhao et al. 2020b); *Micromus angulatus* MK472073; *M. paganus* NC_060415; *Conwentzia sinica* MN200022 (Song et al. 2019); *Semidalis macleodi* MN506226; *Gryposmylus pennyi* MK341582 (Xu et al. 2019); *Osmylus fulvicephalus* MN818867 (Xu et al. 2020); *Thaumatosmylus hainanus* MK408756 (Xu et al. 2019b); *Thyridosmylus langii* KC515397(Zhao et al. 2013); *Euclimacia badia* MF972496; *Eumantispa harmandi* MF972495; *Mantispa japonica* MF972494; *Ditaxis biseriata* FJ859906 (Cameron et al. 2010); *Chrysopa pallens* JX033119 (He et al. 2012); *Chrysoperla externa* KU877169; *C. nipponensis* KX670541; *Apochrysa matsumurae* NC_015095 (Haruyama et al. 2011); *Nothochrysa sinica* NC_015095 (Haruyama et al. 2011); *Polystoechotes punctatus* FJ171325(Beckenbach and Stewart 2009); *Oliarces clara* KT425090 (Wang et al. 2016); *Rapisma zayuanum* KF626447 (Wang et al. 2013); *R. xizangense* KF626446) (Wang et al. 2013); *Balmes birmanus* KT425083 (Wang et al. 2016); *Nymphes myrmeleonoides* KJ461322 (Yan et al. 2014); *Chasmoptera huttii* KT425069 (Wang et al. 2016);.

To understand the evolutionary status of *L. sibiricus*, the mitochondrial genome of 59 species of Neuroptera and four outgroup species of Sialidae were used for phylogeny construction. The maximum likelihood-based phylogenetic tree revelaed (Figure 3) that the mitogenome of *L. sibiricus* is the most closely related to *L. macaronius* with 100% bootstrap support, as shown in Figure 3. Furthermore the phylogenetic tree of the mitochondrial genome of 59 species of Neuroptera revealed that all the owlflies(*Ascaloptynx appendiculatus*, *Suhpalacsa longialata*, *Ogcogaster segmentator*, *Ascalohybris subjacens*, *L. macaronius* and *L. sibiricus*) formed the monophyletic group within the superfamily Myrmeleontoidea.

## Discussion and conclusion

Hypotheses of the exact relationship of the Ascalaphidae and Myrmeleontidae to the other families of the Myrmeleontiformia have changed with time. The paraphyly of Myrmeleontidae, with the previously nested Ascalaphidae, has been supported by multiple phylogenomic studies (Wang et al. 2016, Winterton et al. 2017, Machado et al. 2019). Specifically, Machado et al. (2019) proposed a new classification of Myrmeleontidae, treating Ascalaphidae as a subfamily. In this study, we present the complete mitochondrial genome of *L. sibiricus.* Overall, our findings are consistent with those of Jones (2019) and Machado et al. (2019). However, current molecular data for Ascalaphidae are very limited, especially mitochondrial genome-wide data, which may help resolve the taxonomic inconsistency between the molecular and morphological identification of species in this family more accurately.

In summary, this study is the report of the complete mitochondrial genome of *L. sibiricus*, which is an important species of owlfly. The mitogenome complements the current molecular data for *L. sibiricus* and may contribute to further phylogenetic analyses of Ascalaphidae species and the relationship between Ascalaphidae and the subfamily of Myrmeleontidae.

## Supplementary Material

Supplemental Material

## Data Availability

The data that support the findings of this study are openly available in GenBank at https://www.ncbi.nlm.nih.gov/genbank/, reference number OR571471. The associated BioProject, Bio-Sample numbers, and SRA are PRJNA1027100, SAMN37789662, and SRR26395933, respectively.
