# Supplementary figures and images for: Characterization of the complete mitochondrial genome of the Libelloides sibiricus (Neuroptera, Ascalaphidae)
Source: Mitochondrial DNA B Resour. 2024 Apr 12;9(4):493–9. doi: 10.1080/23802359.2024.2339486 (PMC11018059; doi:10.1080/23802359.2024.2339486)

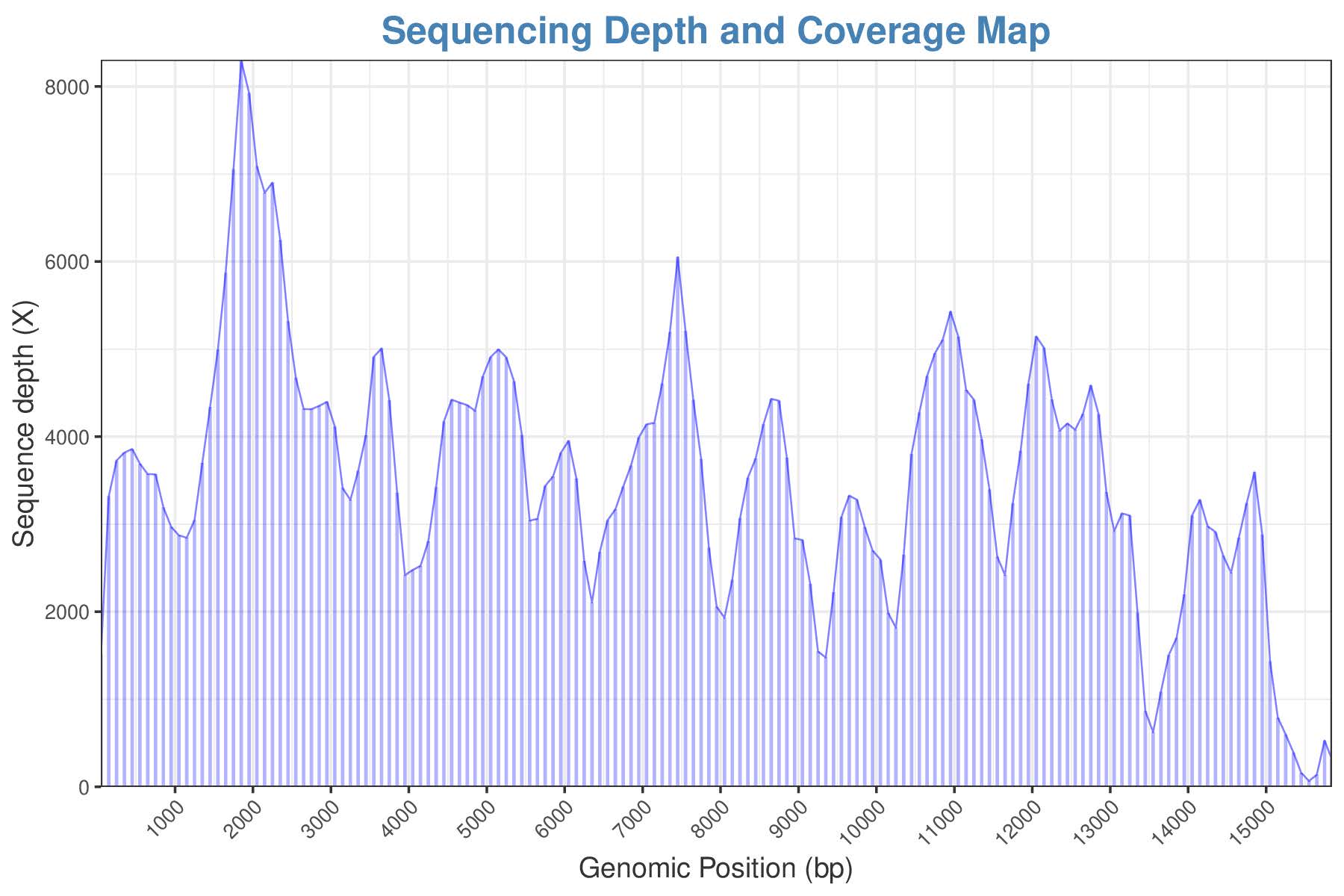

Supplement: Supplemental Material [file TMDN_A_2339486_SM2392.jpg]
